# Correlates of intimate partner violence among adolescents in East Africa: a multi-country analysis

**DOI:** 10.11604/pamj.2021.40.142.23311

**Published:** 2021-11-05

**Authors:** Peter Memiah, Courtney Cook, Caroline Kingori, Leso Munala, Kathryn Howard, Sandra Ayivor, Tristi Bond

**Affiliations:** 1 Division of Epidemiology and Prevention, Institute of Human Virology, University of Maryland School of Medicine, Baltimore Maryland, United States of America,; 2 Biology Department, University of West Florida, Florida, United States of America,; 3 Department of Social and Public Health, College of Health Sciences and Professions, Ohio University Grover Center, Athens OH 45701, United States of America,; 4 Department of Public Health, St. Catherine University, Minnesota, United States of America,; 5 College of Education and Professional Studies, University of West Florida, Florida, United States of America

**Keywords:** Intimate partner violence, adolescents, East Africa

## Abstract

**Introduction:**

intimate partner violence (IPV) is a global concern not only among adults but also adolescents. It has been reported that 35% of adolescent women have ever experienced IPV - occuring more so in non-industrialized countries. This study sought to understand the correlates associated with experiencing IPV among adolescent women between the ages 15 and 24 in five East African countries: Burundi, Kenya, Rwanda, Tanzania, and Uganda.

**Methods:**

this was a secondary analysis of Demographic and Health Survey (DHS) data on adolescent women aged 15-24 years in five East African countries. IPV was measured as a composite variable of emotional, physical, and sexual violence. Other sociodemographic, income, maternal, sexual, knowledge, behavioral, and partner-related variables were included in the analysis.

**Results:**

the prevalence of ever experiencing IPV was 45.1% (n=2380). A higher proportion of women who reported experiencing IPV had their first sexual encounter when they were less than 18 years of age (p<0.001). The adjusted odds ratio (aOR) of experiencing IPV increased almost two times for women who were aged 18-24 years (aOR: 1.7; CI: 1.3-2.3), almost four times (aOR 3.8; CI: 1.7-8.3) for those who had two or more children, and two-fold for women who had ever terminated a pregnancy compared to those who had not (aOR 2.2; CI: 1.0-4.9). Additionally, there was a higher odds (aOR: 1.5 (1.0-2.3)) of experiencing IPV if the respondent believed their husband/spouse´s abuse was justified.

**Conclusion:**

raising early awareness and educating both the young males and females appropriately to mitigate contributing factors to IPV could ensure stable, healthy relationships free of domestic violence in the future.

## Introduction

As violence against adolescent women becomes ubiquitous, 35% globally identify themselves as the victims of intimate partner violence (IPV) with reports emerging from non-industrialized countries that these rates are rising [1,2]. Intimate partner violence (IPV) is a composite variable consisting of physical (slapping or hitting), sexual (forced intercourse), and emotional (threats, intimidation, and controlling behaviors) abuse perpetrated by someone the victim is intimately involved with, typically a husband or partner [3]. Although similar, intimate partner violence is not to be confused with the broader term of domestic violence [4]. IPV is often committed in secrecy as victims may be left with a feeling of guilt or shame while fear of further violence and retribution can prevent victims from seeking help and safety. As stigma and fear conceal IPV, it is thought that the true prevalence of IPV is higher than what is currently reported [5]. Determining the correlates of this violation in human rights is necessary in order to improve the safety and quality of life for adolescent women residing within the East African countries of Burundi, Kenya, Rwanda, Tanzania, and Uganda as IPV becomes more prevalent within the 18 to 24 year age range, a pivotal time in which foundations are set for future wellbeing [6].

Prior studies have found that those who perpetrate intimate partner violence have themselves been victims of IPV or witnessed these behaviors during childhood [6]. This assertion supports the belief that IPV is culturally rooted and a product of sociodemographic characteristics [7]. As adolescent women are influenced by society, inequality, and abuse, IPV becomes accepted and influential [8]. Data from the 2014, 2015, and 2016 Demographic and Health Surveys (DHS) determined prevalence of adolescent IPV among those between the ages of 15 and 24 to be 16.1% in Burundi; 9.6% in Kenya; 19.9% in Rwanda; 12.7% in Tanzania, and 14.9% in Uganda [9-12]. Much of the prior research on IPV has focused on either adult relationships or its occurrence within industrialized nations. When determining the risks faced by adolescent women, specific risks are overlooked causing missed opportunities to prevent IPV during adolescence as well as later in life. In order for programs that are established with the purpose of recognizing and preventing IPV to succeed, specific needs and vulnerabilities of adolescent women must be addressed [13].

In our analysis of IPV against adolescent women, our outcome variable, IPV, was classified as a composite variable of emotional, physical, and sexual violence. Physical violence: push you, shake you, throw something at you, slap you, punch you, kick you, drag you, “beat you up”, try to choke or strangle you, burn you on purpose, threaten you with a knife or any other weapon, or attack you with a knife or any other weapon. Emotional violence: does/did he ever say or do something to humiliate you in front of others? Does he threaten you or someone close to you with harm? Does/did he become jealous or angry if you talk/talked to other men? Sexual violence: forced you to have sexual intercourse when you did not want to, forced you to perform any sexual acts you did not want to, forced or made you have sex with another person. IPV was then measured against the following independent variables to determine whether or not they influenced IPV. Socio-demographic variables include: age, marital status, educational level, religion, and type of place of residence. Income-related variables include: occupational status and wealth index. Maternal-related variables include: number of children and ever terminated a pregnancy. Sexual knowledge and behavioral variables include: sexual debut, recent sexual activity, experienced forced sex, sexual autonomy (constructed variable on whether a partner can refuse sex or ask their partner to use a condom), use of contraceptives, recent sexually transmitted infection (STI), HIV knowledge, number of sexual partners, whether individual received money, gifts, or favors in return for sex, and whether partner is justified in beating the woman (i.e. if the woman believed the husband is justified in beating his wife when she burns the food; women who believe a husband is justified in beating his wife; women who believe a husband is justified in beating his wife when she goes out without telling him). Partner-related variables include: husband/partner´s age and husband/partner´s education. The snapshot data of these indicators in East Africa is shown in Table 1.

**Table 1:** snapshot data of adolescent indicators in East Africa

Country	Year	Population (no.)	Population 15-24 years (no.)	GDP per capita (USD)	Crude birth rate per 1000	Maternal mortality per 100,000 live births	Under 5 mortality per 1,000 live births	Neonatal mortality per 1,000 live births	Sexual violence 15-24 years	Condom use among women 15-24 years
Burundi[1]	2016-2017	10.9 M	2.2 M*	285.7	42.2	712	72	24	16.1%*	3.2%*
Kenya[2]	2014-2015	47.2 M	21.7 M*	1143.1	30.5	362	52	22	9.6%*	15.3%*
Rwanda[3]	2015-2016	11.9 M	4.4 M*	719	32.6	210	50	20	19.9%*	13%*
Tanzania[4]	2015-2016	55.6 M	41.7 M*	867	37.2	398	67	25	12.7%*	25.4%*
Uganda[5]	2015-2016	41.4 M	29.2 M*	580.4	42.1	432	64	27	14.9%*	19.1%*

* indicates data specific to 15-24 age range; M: millions; GDP: gross domestic product

In order to safeguard the futures of the young women of East Africa, systems to prevent and intervene in adolescent IPV are necessary and should be seen as an investment into the future [14]. Intervening in IPV among adolescent women can ultimately lead to the reduction of violence among adults and create early awareness of this human rights violation. With a level of secrecy greater than its adult counterpart, knowledge of the behaviors surrounding adolescent IPV and its true prevalence are still limited.

## Methods

For the purpose of this study, data was derived from the most recent Demographic and Health Surveys (DHS) conducted within the East African countries of Burundi (2016-2017), Kenya (2014), Tanzania (2017), Uganda (2016), and Rwanda (2015). The DHS is a cross-sectional analysis of females and males aged 15-49 years but for the purpose of this study, only data from adolescent women among the ages of 18-24 years was utilized. The Demographic and Health Survey is a household-based, nationally representative, cross-sectional study conducted by ICF Macro/MEASURE DHS on behalf of The National Ministries of Health with financial support from the United States Agency for International Development (USAID) [15]. The DHS program aims to collect data that are comparable amongst separate countries. The DHS program has developed standardized and validated questionnaires and descriptions as to why those questions are being asked of respondents. Approximately every five years, countrywide census data is utilized to determine and select clusters, otherwise known as primary sampling units. Similar to this study, DHS data has previously been used to conduct cross-country analyses. DHS household surveys employ standardized questionnaires and modules for household, women´s and men´s interviews. More extensive details on this data (e.g. sampling criteria and data processing) can be found in the final report of each specific country on the DHS program´s website.

Statistical analysis included a total sample size of 13,165 women aged 15-24 years that had responded to the DHS variable on IPV. The distribution of respondents by country was as follows; Burundi (1,788 adolescent women), Kenya (4,787 adolescent women), Rwanda (567 adolescent women), Tanzania (2,644 adolescent women), and Uganda (3,379 adolescent women). Data derived from DHS was then weighted and analyzed using STATA 14 for Windows. Analysis involved descriptive statistics, specifically frequencies and percentages for all hypothesized correlates of IPV and inferential statistics using Chi-square tests to assess bivariate association among IPV and covariates. Logistic regression analyses were used to assess for associations between covariates and IPV reporting the odds ratios (OR) with respective 95% confidence intervals. Statistical level of significance was evaluated at 5%.

## Results

Table 2 shows the distribution of respondents ever experiencing IPV. The adolescent women were aged between 15-24 years (95.4%, p=0.05). Almost half (53.8%, p<0.001) were single (never in union). A higher majority had primary level education (61.8%, p<0.01). Most of the respondents resided in rural areas (77.3%, p<0.001).

**Table 2:** characteristics of adolescent and young adult females aged 15-24 years who ever experienced intimate partner violence

Variables	Overall, n (%)	Ever experienced IPV		(Pearson chi2)
		Yes, n (%)	No, n (%)	P-value
**Socio-demographic variables**				
**Age in years**				**(3.7) 0.05**
15-<18	241 (4.6)	94 (4.0)	147 (5.1)	
18-24	5034 (95.4)	2285 (96.0)	2749 (94.9)	
**Marital status**				
Never in union	2840 (53.8)	1197 (50.3)	1643 (56.7)	(63.5) < 0.001*
Married	2200 (41.7)	1021 (42.9)	1179 (40.7)	
Separated	154 (4.5)	161 (6.8)	74 (2.6)	
**Level of education**				(65.7) <0.001*
None	724 (13.7)	338 (14.2)	386 (13.3)	
Primary	3262 (61.8)	1577 (66.3)	1685 (58.2)	
Secondary	1194 (22.6)	443 (18.6)	751 (25.9)	
Tertiary	95 (1.9)	21 (0.9)	74 (2.6)	
**Religion**				**(1.11) 0.774**
Catholic	1,373 (38.3)	635 (38.1)	738 (38.5)	
Protestant	1710 (47.7)	789 (47.3)	921 (48.0)	
Muslim	402 (11.2)	194 (11.6)	208 (10.9)	
No religion	100(2.8)	50 (3.0)	50 (2.6)	
**Residence**				
Urban	1198 (22.7)	488 (20.5)	710 (24.5)	(11.9) <0.001*
Rural	4077 (77.3)	1891 (79.5)	2186 (75.5)	
**Income-related variables**				
**Currently working**				(37.7) 0.001*
No	1474 (27.9)	565 (23.8)	909 (31.4)	
Yes	3800 (72.1)	1813 (76.2)	1987 (68.6)	
**Wealth index**				
Poor	2,423 (45.9)	1,190 (50.0)	1,233 (37.7)	(44.2) <0.001*
Middle	1002 (19.0)	467 (19.6)	535 (18.5)	
Rich	1128 (35.07)	722 (30.3)	1128 (42.8)	
**Sexual activity, knowledge, and behavior**				
**Sexual debut in years**				(80.5) < 0.001*
<18	3401 (64.5)	1689 (71.0)	1712 (59.1)	
>18	1874 (35.5)	690 (29.0)	1184 (40.9)	
**Recent sexual activity**				(9.4) <0.001*
Never	4233 (80.3)	1910 (80.3)	2323 (80.2)	
Active	402 (7.6)	157 (6.6)	245 (8.5)	
Not active presently	640 (12.1)	312 (13.1)	328 (11.3)	
**Experienced forced sex**				(74.1) <0.001*
No	4988 (94.6)	2179 (91.6)	2809 (97.0)	
Yes	287 (5.4)	200 (8.4)	87 (3.0)	
**Sexual autonomy**				**(9.6) 0.002**
No	1875 (35.5)	792(33.3)	1083 (37.4)	
Yes	3400 (64.5)	1587 (66.7)	1813 (62.6)	

* indicates p-value level of significance at <0.001

There were statistically significant differences between women who reported to having experienced IPV and those who have not (Table 2). The prevalence of IPV was highest among women who were aged 20-24 years (80%, p=0.001), were married (93.1%, p<0.001), had a primary level of education (66.1%, p<0.001) compared to higher level education, lived in rural areas (79.5%, p<0.001), were of the Catholic faith (34.7%, p=0.011), and identified as poor on the wealth index (50.2%, p<0.001). Based on sexual knowledge and behavior, a higher proportion of women who reported experiencing IPV had their first sexual encounter when they were less than 18 years of age (p<0.001), had no knowledge of HIV (p=0.003), and exhibited sexual autonomy (p=0.001), referring to the role of females regarding decisions concerning how, when and with whom to have sexual relations both within and out of wedlock. A statistically significant association is that most females who report experiencing IPV have never used contraceptives (p=0.766).

An assessment of associations between the prevalence of IPV and husband/partner characteristics shows notable results. Adolescent females who reported that their husband/partner was justified in beating women experienced a higher prevalence of IPV (p<0.001). Additionally, females whose husband/partner had a lower level of education (p<0.001), had a higher prevalence of IPV (Table 2 (suite)).

**Table 2(suite):** characteristics of adolescent and young adult females aged 15-24 years who ever experienced intimate partner violence

Variables	Overall, n (%)	Ever experienced IPV		(Pearson chi2)
		Yes, n (%)	No, n (%)	P-value
**Sexual activity, knowledge, and behavior**				
**Use of contraceptives**				**(11.31) 0.001**
Never used	1882 (35.7)	907 (38.1)	975 (33.7)	
Used or currently using	3393 (64.3)	1472 (61.9)	1921 (66.3)	
**Recent STI (last 12 months)**				(19.5) <0.001*
No	3333 (93.0)	1517(91.0)	1816 (94.7)	
Yes	252 (7.0)	151 (9.0)	101 (5.3)	
**HIV knowledge**				**(4.1) 0.042**
No	1742 (33.0)	738 (31.0)	1004 (34.7)	
Yes	3533 (67.0)	1641 (69.0)	1892 (65.3)	
**Number of sexual partners (excluding husband/spouse)**				(14.8) <0.001*
None	5117 (97.0)	2284 (96.0)	2833 (97.8)	
One or more	158 (3.0)	95 (4.0)	63 (2.2)	
**Received money, gifts or favors in return for sex**				**(4.1) 0.042**
No	527 (96.5)	210 (94.6)	317 (97.8)	
Yes	19 (3.5)	12 (5.4)	7 (2.2)	
**Maternal variables**				
**Number of children**				(134.5) <0.001*
None	798 (15.3)	249 (10.5)	549 (19.0)	
One child	2089 (39.6)	864 (36.3)	1225 (42.3)	
Two or more children	2388 (45.3)	1266 (53.2)	1122 (38.7)	
**Ever terminated a pregnancy**				(25.9) <0.001*
No	4665 (88.4)	2045 (86.0)	2620 (90.5)	
Yes	610 (11.6)	334 (14.0)	276 (9.5)	
**Partner related variables**				
**Husband/partner’s education**				
No education	603(12.0)	281 (12.6)	322 (11.5)	(35.1) <0.001*
Primary	2954 (58.7)	1380 (62.0)	1574 (56.1)	
Secondary	1265 (25.1)	502 (22.5)	763 (27.2)	
Tertiary	209 (4.2)	64 (2.9)	145(5.2)	
**Husband or partner’s age in years**				**(0.03) 0.861**
<24	1449 (29.0)	636 (28.9)	813 (29.1)	
30-39	3546 (71.0)	1566 (71.1)	1980 (70.9)	
**Does the respondent believe it is justified for a man to beat a woman?**				(7.6) <0.001*
No	2122 (40.2)	738 (31.0)	1384 (47.8)	
Yes	3153 (59.8)	1641 (69.0)	1512 (52.2)	

* indicates p-value level of significance at <0.001

Results from multivariate logistic regression analysis show that the odds of experiencing IPV increases almost two times for women who are aged 18-24 years (OR 1.7; 95% CI 1.3-2.3; p=0.047), almost four times for those who have two or more children (OR 3.8; 95% CI 1.7-8.3; p=0.001), and twofold for females who have ever terminated a pregnancy (OR 2.2; 95% CI 1.0-4.9; p=0.042) compared to those who have not (Table 3). Additionally, the odds of experiencing IPV increases 10 times among females whose husband/partner had no formal education (OR 10.2; 95% CI 2.8-37.6; p<0.001) and almost two times among females who believe that it is justified for a man to beat a woman (OR 1.5; 95% CI 1.0-2.3; p=0.028) (Table 3 (suite)).

**Table 3:** unadjusted and adjusted logistic regression analysis of the prevalence of intimate partner violence

Variables	Unadjusted		Adjusted	
	OR (95% CI)	P-value	OR (95% Cl)	P-value
**Socio-demographic variables**				
**Age**				
15-<18	REF			
18-24	1.3 (1.1-1.4)	0.04	1.7 (1.3-2.3)	0.047
**Marital status**				
Never in union	REF			
Married	1.2 (1.1-1.3)	0.002	0.2 (0.1-1.3)	0.348
Separated	2.9 (2.2-3.9)	<.001*	2.9 (0.9-9.1)	0.057
**Level of education**				
None	3.1 (1.8-5.1)	<0.001*	1.3 (1-1.7)	0.101
Primary	3.3 (2.0-5.4)	<0.001*	1.2 (0.9-1.6)	0.231
Secondary	2.1 (1.2-3.4)	<0.001*	0.6 (0.2-1.7)	0.34
Tertiary	REF			
**Residence**				
Urban	REF			
Rural	1.2 (1.1-1.4)	<0.001*	1.8 (0.5-1.9)	0.425
**Income-related variables**				
**Currently working**				
No	1.4 (1.3-1.7)	<0.001*	1.3 (0.9-1.9)	0.14
Yes	REF			
**Wealth index**				
Poor	1.5 (1.1.3-1.7)	<0.001*	1.2 (0.7-1.9)	0.747
Middle	1.3 (1.1-1.6)	<0.001*	1.3 (0.7-2.3	0.351
Rich	REF			
**Maternal variables**				
**Number of children**				
None	REF			
1 child	1.5 (1.3-1.8)	<0.001*	1.9 (0.9-4.2)	0.075
2 or more children	2.5 (2.1-2.9)	<0.001*	3.8 (1.7-8.3)	0.001
**Ever terminated a pregnancy**				
No	REF			
Yes	1.6 (1.3-1.8)	<0.001*	2.2 (1.0-4.9)	0.042

* Indicates p-value level of significance at <0.001

**Table 3(suite):** unadjusted and adjusted logistic regression analysis of the prevalence of intimate partner violence

Variables	Unadjusted		Adjusted	
	OR (95% CI)	P-value	OR (95% Cl)	P-value
**Sexual activity, knowledge, and behavior**				
**Sexual debut**				
<18	1.6 (1.5-1.9)	<0.001*	1.1 (0.7-1.8)	0.556
≥18	REF			
**Recent sexual activity**				
Never	REF			
Active	0.8 (0.6-0.9)	0.019	0.4 (0.2-0.9)	0.038
Not presently active	1.2 (0.9-1.4)	0.076	1.2 (0.7-2.1)	0.481
**Experienced forced sex**				
No	REF			
Yes	3.0 (2.3-3.8)	<0.001*	1.4 (0.7-3.3)	0.323
**Sexual autonomy**				
No	REF			
Yes	1.2 (1.1-1.3)	0.002	1.2 (0.4-3.4)	0.69
**Contraceptive use**				
Never used	REF			
Used or currently use	1.5 (1.3-1.9)	<0.001*	1.3 (0.8-1.9)	0.216
**Recent STI (last 12 months)**				
No	REF			
Yes	1.8 (1.4-2.4)	<0.001*	1.2 (0.4-.3.5)	0.69
**HIV knowledge**				
No	1.2 (1.1-1.3)	0.004	1.1 (0.8-1.5)	0.437
Yes	REF			
**Number of sexual partners (excluding husband/spouse)**				
None	REF			
One or more	1.9 (1.4-2.6)	<0.001*	1.0 (0.3-3.2)	0.998
**Receipt of gifts, money, or favors in return for sex**				
No	REF			
Yes	2.6 (1.6-6.7)	0.049	2.2 (0.7-6.4)	0.138
**Partner-related variables**				
**Husband/partner's education**				
None	2.0 (1.4-2.7)	<0.001*	10.2 (2.8-37.6)	<0.001*
Primary	2.0 (1.5-2.7)	<0.001*	2.6 (1.1-6.5)	0.037
Secondary	1.5 (1.1-2.0)	0.013	2.1(0.8-5.2)	0.101
Tertiary	REF			
**Does the respondent believe it is justified for a man to beat a woman?**				
No	REF			
Yes	2.0 (1.8-2.3)	<0.001*	1.5 (1.0-2.3)	0.028

* indicates p-value level of significance at <0.001

## Discussion

Intimate partner violence (IPV) is a pervasive public and global health issue. It can lead to health issues such as chronic pain, mental disorders, suicide ideation and risky sexual behavior as well as low socio-economic status [3,8,16,17]. Our study findings found a prevalence of 45.1% which is within the range of 13 to 61 percent lifetime prevalence of IPV as reported by the World Health Organization (WHO) multi-country study on women´s health and domestic violence [18] and aligns with other published studies [19].

Among the data collected from adolescent women that had experienced intimate partner violence, 12.6% of husbands/partners had received no formal education, and 62% had attained only primary level education. The WHO has determined that low educational attainment for males and females, or disparities in educational attainment, is a frequent contributing factor to IPV [20]. In an effort to address these disparities, programs in low-income countries which promote education and skilled trades can promote future financial stability. In settings where children and adolescents are attending educational programs, classroom-based learning initiatives can teach adolescents safe dating skills and influence behaviors related to violence [20]. Participants whose partners had lower than tertiary level of education were 10 times more likely to experience IPV. Documented evidence reports that men who abuse their partners lack socially acceptable coping strategies [21]. Thus, it is assumed that educated men are less likely to be abusive because they have been exposed to alternative ways of dealing with frustrations and are presumed to be economically stable enough to avoid pitfalls associated with violence, such as gambling, sexual dysfunction, and substance abuse [21-24]. To that end, it is critical to develop interventions targeting men, particularly those who have low levels of education, and provide them with skills and knowledge that enhance their coping strategies and aid in healthy relationships.

Many studies have found that females who are the victims of IPV are more likely to report events such as unintended pregnancy and termination of a pregnancy. Although fewer in number, additional studies found that females who had ever terminated a pregnancy, most of which were planned pregnancies, were more likely to report IPV [25]. Reasons for wanting to terminate their pregnancies ranged from coercion by their spouses to individual efforts to regain their reproductive freedom and sexual autonomy [1].

In our study adolescent women that had two or more children had experienced intimate partner violence. During a study within the Amazon region, later published in the journal Nature Human Behavior, it was found that women in this region who were the victims of intimate partner violence were 10 to 15% more likely to have a child compared to other women their age, the average age of these women was 18. Reasons for the increase in reported pregnancies and rates of intimate partner violence as high as 85% can be the result of culturally rooted violence where men assert their will over their spouses. The study suggests that rather than an increase in the number of children being a risk factor for IPV, IPV is a cause of larger family size [26].

Approximately 60% of adolescent women reported that they believed their husband/spouse´s acts of intimate partner violence were justified. Similar results were found during a study in Ethiopia. During the Ethiopian study, female respondents were given five separate partner violence scenarios and asked to give their opinion. DHS questionnaire data was also collected from respondents. All 64.9% of the respondents reported that the husbands were justified in beating their spouses in the scenarios presented [5]. Global data from multiple sources, has determined that IPV is normative in many settings, with both men and women expressing support for this culturally-rooted phenomena. The acceptance of such violence is frequently supported by the belief of men´s need to ‘discipline´ women for various behaviors often related to gender roles and expectations regarding expected female behavior [27].

It is worth noting that in the bivariate analyses, variables related to sexual activity, knowledge, and behavior - such as sexual debut, forced sex, sexual autonomy, contraceptive use, sexually transmitted infections, justified wife beating. HIV knowledge of one’s sexual partners was significantly associated with IPV. Regarding HIV knowledge, the probability of IPV against women increases HIV incidence and decreases post-disclosure of HIV status, medication adherence, and viral suppression. This may serve as a deterrent for the general uptake of HIV services and prevention strategies among peers who are fearful of experiencing IPV from disclosing their status [28,29]. These findings are supported by a 2014 study which found IPV was likely to occur among adolescent women who: had experienced sexual abuse in childhood, had partners concurrently engaged, experienced forced or unwanted sexual intercourse, and accepted spousal abuse [2,19,30].

Predictors of IPV were found to be associated with the following variables: low levels of partner education, pregnancy termination, never having an active sex life, having two or more children, and a belief that a husband/partner is justified in beating women. Such findings speak volumes to the accepted social norms in a given society and the pervasive nature in which they are manifested at such a young age among adolescent women. Social norms are the informal rules of behavior that dictate what is standard in a particular social context and may influence IPV or related behavior [1]. These findings support the importance of developing prevention strategies early in this target group to minimize the occurrence of IPV in adulthood. Particularly, there is a need to increase prevention efforts that reduce childhood abuse, such as home visitations and parenting initiatives, that will support improving parent-child relationships.

**Limitations:** this study relied on DHS data which is retrospective in nature. Because of this, potential limitations include reporting and recall bias due to potential memory deficits. IPV exposure may be under-reported in the study region due to social stigma, which limits the generalizability of these findings. Given the cross-sectional nature of DHS data, we could only examine associations; we used Chi-square tests for bivariate associations among IPV and covariates and logistic regression analyses (CI 95%) to analyze associations among IPV and covariates. We also deliberately did not conduct any country comparisons as this was not the objective of our study. Despite these potential limitations, the study findings provide a current, culturally relevant idea of covariates associated with adolescent IPV risk. These findings should be used to facilitate contextualized interventions across East African countries.

## Conclusion

The pervasive nature of IPV among adolescent women is disconcerting. In East Africa, there is a critical need to develop targeted policies and health services that will resonate with adolescent women. In addition, the government and health partners should integrate tailored strategies into existing programs, such as family planning and male involvement programs.

**Ethics approval and consent to participate:** the study used secondary data from measure DHS and did not require ethical approval.

**Availability of data and materials:** DHS data and reports are available upon request from the corresponding author.

### What is known about this topic

- One of the main Sustainable Development Goals (SGDs) set out in the United Nations' 2030 agenda seeks to “achieve gender equality and empower all women and girls”;
- Since the International Conference on Population and Development in Cairo in 1994, there has been increasing interest in reducing intimate partner violence (IPV) and promoting gender equality, particularly for sexual and reproductive health (United Nations, 1994);
- The research base for the impact of adolescents experiencing IPV on health-related outcomes continues to be mixed; at the same time, evidence concerning IPV in East Africa remains relatively unexplored.

### What this study adds

- Our study addresses a major critical area - IPV among a vulnerable age group - which is in line with the prevention of violence for the realization of the SDG goal 5;
- We report data from five East African countries, and our study findings provide the burden of IPV as well as new evidence on factors associated with IPV in adolescents;
- Our study findings aid in the development of targeted initiatives that promote adolescent health, particularly among young women; the pervasive nature of IPV among adolescent women is disconcerting, and there is a critical need to develop targeted policies and health services that will resonate with adolescent women in East Africa.
